# Bilateral multifocality is an independent predictor of patients’ outcome in Middle Eastern papillary thyroid carcinoma

**DOI:** 10.3389/fendo.2022.1060301

**Published:** 2023-01-04

**Authors:** Sandeep Kumar Parvathareddy, Abdul K. Siraj, Padmanaban Annaiyappanaidu, Nabil Siraj, Saif S. Al-Sobhi, Fouad Al-Dayel, Khawla S. Al-Kuraya

**Affiliations:** ^1^ Human Cancer Genomic Research, Research Center, King Faisal Specialist Hospital and Research Center, Riyadh, Saudi Arabia; ^2^ Department of Surgery, King Faisal Specialist Hospital and Research Center, Riyadh, Saudi Arabia; ^3^ Department of Pathology, King Faisal Specialist Hospital and Research Centre, Riyadh, Saudi Arabia

**Keywords:** papillary thyroid carcinoma, multifocality, bilateral, recurrence-free survival, prognosis

## Abstract

**Background:**

Tumor multifocality is frequently seen in Papillary thyroid carcinoma (PTC). However, few studies have analysed the impact of bilateral multifocality in PTC. The incidence of bilateral multifocality, its clinico-pathological associations and prognostic impact in PTC from Middle Eastern ethnicity remains unestablished.

**Methods:**

We retrospectively evaluated 1283 patients who underwent total thyroidectomy for PTC. Bilateral and unilateral multifocality were decided based on the final pathology result. Primary outcome was recurrence free survival (RFS). Risk factors for bilateral multifocality were analyzed by multivariate logistic regression analysis.

**Results:**

Multifocal PTC was found in 54.3% (697/1283) of patients. Among the 697 multifocal PTCs, 210 patients (30.1%) had unilateral multifocal PTC and 487 patients (69.9%) had bilateral multifocality. Bilateral multifocality was significantly associated with older age at diagnosis (p = 0.0263), male gender (p = 0.0201), gross extrathyroidal extension (p = 0.0332), larger primary tumor size (>4cm; p = 0.0002), lateral lymph node metastasis (p = 0.0008), distant metastasis at diagnosis (p = 0.0195) and recurrence (p = 0.0001). Bilateral multifocality was also found to be an independent predictor of RFS (Hazard ratio = 1.60; 95% Confidence Interval = 1.05 – 2.55; p = 0.0300). Multivariate logistic regression analysis demonstrated tumor diameter >4cm to be the only independent risk factors for bilaterality in multifocal PTC (Odds ratio = 1.86; 95% Confidence Interval = 1.13 – 3.07; p = 0.0155).

**Conclusions:**

Incidence of bilateral multifocality is high in Middle Eastern PTC. Tumor diameter >4cm can be considered as a predictive factor for bilateral multifocal PTC. Bilateral multifocality appears to be an important prognostic factor for PTC and an independent predictor of RFS. Therefore, patients with bilateral multifocal PTC may benefit from more frequent follow-up to identify recurrences earlier.

## 1 Introduction

Papillary thyroid carcinoma (PTC) is the most common endocrine malignancy (1). Its incidence has increased rapidly over the last two decades (2–4). PTC has become the second most prevalent cancer (after breast cancer) among women in Saudi Arabia (5). Although the prognosis of PTC is excellent, up to 20% experience loco-regional recurrence, which might lead to worse clinical outcomes (6–8). The standard treatment of PTC is thyroidectomy followed by selective radioiodine ablation and/or thyroxine therapy (9, 10). However, recent evidence suggests that use of more conservative approaches as well as radioiodine ablation, especially in low risk PTC, are equally effective (11–14). Hence, these treatment approaches, when used in risk defined patients, can reduce the disease recurrence and improve patients’ outcome. There are several clinico-pathological factors affecting the risk of recurrence and prognosis in PTC patients. Most of these factors have been included in the risk stratification system proposed by the American Thyroid Association (ATA) (9). Moreover, accurate risk stratification is vital in helping define the appropriate therapeutic approach of PTC.

PTC with multifocality frequently arise in thyroid gland and has been considered as prognostic marker in PTC (15–17). Several risk stratification systems have addressed the prognostic role of multifocality in PTC including the latest ATA, the European Thyroid Association Guidelines (ETA) and consensus report of European Society of Endocrine Surgeons (9, 18, 19). However, several recent reports debate the impact of multifocality on PTC prognosis and recurrence (20–22). Although many studies have investigated bilateral multifocality, association with clinicopathological factors and prognostic value, the results are still controversial (23–25), which contributes to uncertainty in managing PTC patients with bilaterality multifocality. In addition, the incidence, clinico-pathological associations and prognostic impact of multifocality and bilaterality remained unexplored in PTC of Middle Eastern ethnicity.

Identifying predictive markers of bilateral multifocality and its clinico-pathological associations as well as clinical impact on patients’ prognosis could guide surgeons and oncologists to determine the appropriate treatment for PTC patients. In this retrospective study we investigated the incidence of multifocality in PTC from Middle Eastern ethnicity and its clinico-pathological associations. Moreover, we aimed to determine whether bilateral multifocality can predict patients’ prognosis in this ethnicity.

## 2 Materials and methods

### 2.1 Patient selection

One thousand five-hundred and fifteen consecutive unselected PTC patients diagnosed between 1988 and 2018 at King Faisal Specialist Hospital and Research Centre (Riyadh, Saudi Arabia) were available to be included in the study. Only those patients with information on tumor focality who underwent total thyroidectomy were included in the study. A total of 1283 PTC cases were available to be included in the study. Of these, 697 patients had multifocal PTCs and were included for final analysis. Cases were identified based on clinical history followed by fine needle aspiration cytology for confirmation. The Institutional Review Board of the hospital approved this study and the Research Advisory Council (RAC) provided waiver of consent under project RAC # 221 1168 and # 2110 031.

### 2.2 Clinico-pathological data

Baseline clinico-pathological data were collected from case records and have been summarized in **Table 1**. All patients included in this study underwent total thyroidectomy. Central neck dissection alone was performed in 9.2% (64/697), lateral neck dissection alone was performed in 2.0% (14/697) and both central as well as lateral neck dissection was performed in 81.5% (568/697) of patients. Central and lateral lymph node dissections were performed in accordance with the 2015 American Thyroid Association guidelines (9), which recommends central and lateral lymph node dissection when clinically positive lymph nodes are found pre-operatively (i.e. by ultrasound or fine needle aspiration cytology). Tumor focality was assessed post-operatively on histopathological examination. Presence of two or more tumor foci was classified as multifocal. Multifocal tumors were further sub-grouped as unilateral multifocal and bilateral multifocal PTCs, depending on whether all tumor foci were located in a single lobe or they were seen in both lobes. Based on the 2015 American Thyroid Association (ATA) guidelines, tall cell, hobnail, columnar cell, diffuse sclerosing and insular variants were classified as aggressive variants, whereas classical and follicular variants were classified as non-aggressive variants (9) for the purpose of multivariate analysis. Staging of PTC was performed using the eighth edition of American Joint Committee on Cancer (AJCC) staging system (26). Only structural recurrence (local, regional or distant) was considered for analysis. Recurrence was defined as any newly detected tumor (local or distant) or metastatic regional lymph node (LN) based on ultrasound and/or imaging studies in patients who had been previously free of disease following initial treatment. Hashimoto’s thyroiditis was diagnosed when the typical histopathological features of lymphoplasmacytic infiltration, a germinal center formation, follicular destruction, a Hurthle cell change, and variable degrees of fibrosis were noted (27).

**Table 1:** Clinico-pathological characteristics of multifocal papillary thyroid carcinomas.

	Total	
	No.	%
**Total**	697	
Age at surgery (years)		
Median (range)	38.2 (6.0 – 89.0)	
< 55	563	80.8
≥ 55	134	19.2
Gender		
Male	176	25.3
Female	521	74.7
Histologic subtype		
Classical variant	445	63.8
Follicular variant	93	13.4
Tall cell variant	83	11.9
Other variants	76	10.9
Tumor laterality		
Unilateral	210	30.1
Bilateral	487	69.9
Extrathyroidal extension		
Absent	340	48.8
Microscopic	292	41.9
Gross	65	9.3
Lymphovascular invasion		
Lymphatic invasion only	26	3.7
Vascular invasion only	58	8.3
Both lymphatic and vascular invasion	162	23.3
None	451	64.7
Tumor size		
1-2cm	243	34.8
2-4cm	287	41.2
>4cm	167	24.0
Regional LN metastasis		
N0	241	34.6
N1a	131	18.8
N1b	274	39.3
Nx	51	7.3
pM		
M0	652	93.5
M1	45	6.5
TNM Stage		
I	563	82.1
II	86	12.4
III	10	1.4
IV	28	4.1
*BRAF* mutation		
Present	361	51.8
Absent	215	30.8
Unknown	121	17.4
Recurrence		
Yes	143	20.5
No	554	79.5
Hashimoto’s thyroiditis		
Present	182	26.1
Absent	515	73.9

### 2.3 *BRAF* mutation analysis

*BRAF* mutation data was assessed in our laboratory by Sanger sequencing and has been published by us previously (28).

### 2.4 Follow-up and study endpoint

Radioactive iodine (I-131) therapy was given to 83.2% (1068/1283) of PTC patients in our cohort. All ATA high- and intermediate-risk patients were offered I-131 therapy, whereas low-risk patients with lymph node metastasis also received I-131 therapy. Post-operative serum thyroglobulin levels were analyzed at 6 months and 1 year after surgery. 6.5% (84/1283) of patients showed an elevated post-operative serum thyroglobulin level (> 2ng/ml). Tumor recurrences were detected by serum thyroglobulin levels, whole body scan, ultrasound or CT scans performed during regular follow-up visits. Recurrence-free survival (RFS) was defined as the time (in months) from date of initial surgery to the occurrence of any tumor recurrence (local, regional or distant). In case of no recurrence, date of last follow-up was the study endpoint for RFS.

### 2.5 Statistical analysis

The associations between clinico-pathological variables and tumor focality was performed using contingency table analysis and Chi square tests. Mantel-Cox log-rank test was used to evaluate RFS. Survival curves were generated using the Kaplan-Meier method. Cox proportional hazards model and logistic regression were used for multivariate analysis. Two-sided tests were used for statistical analyses with a limit of significance defined as p value < 0.05. Data analyses were performed using the JMP14.0 (SAS Institute, Inc., Cary, NC) software package.

## 3 Results

### 3.1 Patient and tumor characteristics

Median age of the entire cohort was 38.2 years (range: 6 – 89 years), with a male: female ratio of 1:3. Majority of the tumors were of classical variant (63.8%; 445/697). 24.0% (167/697) of multifocal PTCs were larger than 4cm in largest diameter. Regional LN metastasis was noted in 58.1% (405/697) of cases and distant metastasis was present at diagnosis in 6.5% (45/697). *BRAF* mutation data was available in 576 cases, with mutation being present in 62.7% (361/576) of multifocal PTCs. 20.5% (143/697) of patients developed tumor recurrence. (**Table 1**). The median follow-up was 8.2 years (range = 1.0 – 30.1 years). Recurrences were noted at a median of 3.2 years after initial diagnosis (range = 1 – 17.4 years).

### 3.2 Clinico-pathological associations of bilateral multifocal tumors in PTC

Of the 697 multifocal PTCs included in our study, 69.9% (487/697) were bilateral and 30.1% (210/697) were unilateral. Bilateral multifocality was significantly associated with older age at diagnosis (p = 0.0263), male gender (p = 0.0201), gross extrathyroidal extension (p = 0.0332), larger primary tumor size (>4cm; p = 0.0002), lateral lymph node metastasis (p = 0.0008) and distant metastasis at diagnosis (p = 0.0195) (**Table 2**). Importantly, we found bilateral multifocality to be significantly associated with tumor recurrence (p = 0.0001), with 24.2% (118/487) of bilateral multifocal tumors showing recurrence compared to 11.9% (25/210) of unilateral multifocal PTCs (**Table 2**).

**Table 2:** Clinico-pathological associations of unilateral and bilateral multifocal papillary thyroid carcinomas.

	Unilateral Multifocal		Bilateral Multifocal		p value
	No.	%	No.	%	
**Total**	210	30.1	487	69.9	
Age at surgery (years)					
< 55	180	85.7	383	78.6	0.0263
≥ 55	30	14.3	104	21.4	
Gender					
Male	41	19.5	135	27.7	0.0201
Female	169	80.5	352	72.3	
Histologic subtype					
Classical variant	132	62.9	313	64.3	0.5799
Follicular variant	28	13.3	65	13.3	
Tall cell variant	30	14.3	53	10.9	
Other variants	20	9.5	56	11.5	
Extrathyroidal extension					
Absent	110	52.4	230	47.2	0.0332
Microscopic	89	42.4	203	41.7	
Gross	11	5.2	54	11.1	
Lymphovascular invasion					
Present	74	35.2	172	35.3	0.9838
Absent	136	64.8	315	64.7	
Tumor size					
1-2cm	75	35.7	168	34.5	0.0002
2-4cm	104	49.5	183	37.6	
>4cm	31	14.8	136	27.9	
Regional LN metastasis					
N0	88	43.6	153	34.5	0.0008
N1a	50	24.7	81	18.2	
N1b	64	31.7	210	47.3	
pM					
M0	203	96.7	449	92.2	0.0195
M1	7	3.3	38	7.8	
TNM Stage					
I	183	87.1	387	80.0	0.1092
II	19	9.1	67	13.8	
III	3	1.4	7	1.5	
IV	5	2.4	23	4.7	
*BRAF* mutation					
Present	104	60.5	257	63.6	0.4755
Absent	68	39.5	147	36.4	
Recurrence					
Yes	25	11.9	118	24.2	0.0001
No	185	88.1	369	75.8	
Hashimoto’s thyroiditis					
Present	56	26.7	126	25.9	0.8269
Absent	154	73.3	361	74.1	

### 3.3 Prognostic significance of bilateral multifocality in PTC

**Figure 1:**
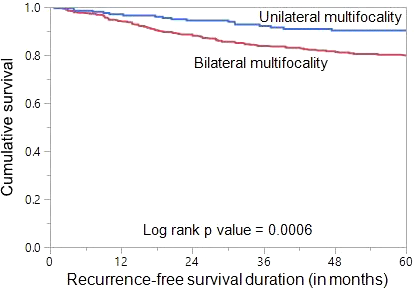
Recurrence-free survival (RFS) in multifocal papillary thyroid carcinoma (PTC). Kaplan-Meier survival curve showing shorter RFS in bilateral multifocal PTC patients compared to those with unilateral multifocal PTC (p = 0.0006).

Since we found bilateral multifocal PTCs to be associated with tumor recurrence, we analysed the prognostic significance of bilateral multifocality with regards to RFS. Using Mantel Cox log rank test, we found that bilateral multifocality was associated with shorter RFS (p = 0.0006) (**Figure 1**). On multivariate analysis using Cox proportional hazards model, bilateral multifocality was found to be an independent predictor of RFS (Hazard ratio = 1.60; 95% Confidence Interval = 1.05 – 2.55; p = 0.0300) (**Table 3**).

**Table 3:** Cox proportional hazards model for predictors of recurrence-free survival (RFS) in multifocal PTC.

	Recurrence-free survival			
	Univariate		Multivariate	
Clinico-pathological variables	HR(95% CI)	p value	HR(95% CI)	p value
Age				
≥ 55 years (vs. < 55 years)	3.23 (2.26 – 4.55)	< 0.0001	2.41 (1.62 – 3.52)	< 0.0001
Gender				
Male (vs. Female)	1.72 (1.21 – 2.42)	0.0027	1.24 (0.86 – 1.78)	0.2509
Histology				
Aggressive variants (vs. non-aggressive variants)	1.39 (0.93 – 2.05)	0.1096		
Tumor laterality				
Bilateral (vs. Unilateral)	2.10 (1.39 – 3.31)	0.0003	1.60 (1.05 – 2.55)	0.0300
Extrathyroidal extension				
Absent	Reference		Reference	
Microscopic	2.17 (1.48 – 3.25)	< 0.0001	1.51 (1.00 – 2.31)	0.0500
Gross	4.81 (2.99 – 7.68)	< 0.0001	1.50 (0.84 – 2.64)	0.1687
Lymphovascular invasion				
Present (vs. Absent)	1.28 (0.89 – 1.82)	0.1777		
Tumor size				
1-2cm	Reference		Reference	
2-4cm	1.42 (0.92 – 2.23)	0.1122	1.44 (0.92 – 2.30)	0.1085
>4cm	3.07 (2.01 – 4.78)	< 0.0001	2.41 (1.57 – 3.78)	< 0.0001
Regional LN metastasis				
Present (vs. absent)	2.33 (1.58 – 3.59)	< 0.0001	1.71 (1.12 – 2.69)	0.0117
Distant metastasis				
Present (vs. absent)	6.13 (3.99 – 9.10)	< 0.0001	2.99 (1.79 – 4.85)	< 0.0001

HR, Hazard ratio; CI, Confidence interval; LN, Lymph node.

Tall cell, hobnail, columnar cell, diffuse sclerosing and insular variants were classified as aggressive variants, whereas classical and follicular variants were classified as non-aggressive variants.

### 3.4 Clinico-pathological factors predicting bilateral multifocality

Next, we sought to determine the clinico-pathological features that could predict bilateral multifocality in PTCs. On univariate analysis using logistic regression model, we found older at diagnosis, male gender, gross extrathyroidal extension, larger tumor size (>4cm), regional lymph node metastasis and distant metastasis at diagnosis to be significantly associated with bilateral multifocality. We included these significant clinico-pathological parameters in a multivariate logistic regression model and found that only tumor size >4cm (Odds ratio = 1.86; 95% Confidence Interval = 1.13 – 3.07; p = 0.0155) was an independent predictor for bilateral multifocality in PTCs (**Table 4**).

**Table 4:** Multivariate analysis using logistic regression model for predictors of bilateral multifocality in PTC.

	Bilateral multifocal PTC			
	Univariate		Multivariate	
Clinico-pathological variables	OR(95% CI)	p value	OR(95% CI)	p value
Age				
≥ 55 years (vs. < 55 years)	1.63 (1.05 – 2.54)	0.0309	1.35 (0.81 – 2.23)	0.2474
Gender				
Male (vs. Female)	1.58 (1.07 – 2.35)	0.0230	1.32 (0.87 – 2.00)	0.1911
Histology				
Aggressive variants (vs. non-aggressive variants)	0.92 (0.63 – 1.35)	0.6803		
Extrathyroidal extension				
Absent	Reference		Reference	
Microscopic	1.09 (0.78 – 1.53)	0.6132	0.97 (0.67 – 1.42)	0.8860
Gross	2.35 (1.18 – 4.67)	0.0149	1.62 (0.77 – 3.42)	0.2040
Lymphovascular invasion				
Present (vs. Absent)	1.00 (0.72 – 1.41)	0.9838		
Tumor size				
1-2cm	Reference		Reference	
2-4cm	0.79 (0.55 – 1.13)	0.1929	0.82 (0.56 – 1.20)	0.3156
>4cm	1.96 (1.22 – 3.15)	0.0056	1.86 (1.13 – 3.07)	0.0155
Regional LN metastasis				
Present (vs. absent)	1.47 (1.04 – 2.06)	0.0269	1.37 (0.95 – 1.98)	0.0909
Distant metastasis				
Present (vs. absent)	2.45 (1.08 – 5.59)	0.0325	1.64 (068 – 3.96)	0.2711

OR, Odds ratio; CI, Confidence interval; LN, Lymph node.

Tall cell, hobnail, columnar cell, diffuse sclerosing and insular variants were classified as aggressive variants, whereas classical and follicular variants were classified as non-aggressive variants.

## 4 Discussion

The optimal clinical management of PTC depends on appropriate clinico-pathological risk stratification. Therefore, accurately evaluating the clinico-pathological risk factors is important in achieving the balance between higher therapy benefit and lower adverse complications. Multifocality as prognostic marker remains a highly controversial subject (15, 20–23, 29–33). These conflicting results in the literature could be attributed to the clinical behavior difference between unilateral and bilateral tumors. Even among the few studies that compared unilateral and bilateral multifocal PTC, the prognostic impact of multifocality remains inconsistent. While Sefika et al. (24) could not find association between bilateral involvement in multifocal PTC and prognosis, Jiu et al. (25) found both bilaterality and multifocality to be associated with aggressive tumor but multifocality only was associated with increased risk of recurrence. Another study by Woo et al. (34) found no significant difference between unifocal and multifocal PTC in terms of prognosis, but reported that multifocality was an independent risk factor for recurrence in a cohort of more than 2000 PTCs. In this study, we evaluated for the first time, the incidence of multifocality and clinical impact of unilateral and bilateral subgroups in a large cohort of PTCs from Middle Eastern ethnicity.

In our cohort, multifocality was observed in 54.3% of patients, which is comparable with what has been reported in literature (15, 22, 23, 35). The rate of bilateral multifocal PTC was 69.9% (487/697). The incidence reported in literature ranges from 13 – 71% (23, 24, 36–38). Results of our study show that bilaterality in multifocal PTC is associated with aggressive clinico-pathological markers such as older age, male gender, gross extrathyroidal extension, larger tumor size, locoregional and distant metastasis. Previous studies have also reported an association between multifocality and aggressive clinico-pathological characteristics (23, 24, 39–41).

Interestingly, bilateral multifocality was significantly associated with unfavorable clinical outcomes, being an independent predictor of RFS. The prognostic value of multifocality was significant in bilateral multifocality subgroup compared to unilateral multifocal PTC subgroup, showing an approximately two-fold increased risk of recurrence. Therefore, this study has identified that bilateral multifocality is a risk factor for recurrence after total thyroidectomy in Middle Eastern PTC patients. This is consistent with a previous report which showed that bilateral multifocality was an independent risk factor for neck recurrence as well as distant metastasis (42).

Given the strong prognostic impact of bilateral multifocality, we further evaluated if there are any predictive markers of bilateral multifocal PTCs. We found that tumor diameter of >4cm was the only independent predictor for bilaterality in multifocal PTCs. Previous studies have identified number of tumor foci, TSH level, and *BRAF* mutation as predictors of bilateral multifocal disease (24, 43). However, number of tumor foci and preoperative TSH level data were not used as variables in this cohort due to lack of documented data on these variables. *BRAF* mutation was not associated with bilateral multifocal disease in this study.

The present study supported the finding of previous studies that multifocality is associated with aggressive disease (22, 42, 43). Bilateral multifocality has a strong association with advanced disease and more metastatic potential as well as increased risk of recurrence. In addition, bilateral multifocality plays an important role in patient prognosis and in predicting RFS in PTC patients.

Our study has a few limitations. Firstly, this is a retrospective single institute study which includes only PTC patients from Saudi Arabia and hence selection bias is inevitable. The results of this study should be interpreted with caution and generalizability of our results to other ethnicities needs to be validated in large studies from different ethnicities. Secondly, data on the number of tumor foci are not routinely recorded in our histopathological reports and hence we were unable to incorporate this interesting data in our current study.

In conclusion, bilateral multifocality is high in Middle Eastern PTC and is associated with aggressive clinico-pathological markers. Tumor diameter >4cm was predictive of bilateral multifocality in PTC. Bilateral multifocality appears to be an important prognostic factor for PTC and an independent predictor of RFS. Therefore, patient with bilateral multifocal PTC may benefit from more frequent follow-up to identify recurrences earlier.

## Data availability statement

The original contributions presented in the study are included in the article/supplementary material. Further inquiries can be directed to the corresponding author.

## Ethics statement

The studies involving human participants were reviewed and approved by Research Advisory Council, King Faisal Specialist Hospital and Research Centre. Written informed consent from the participants’ legal guardian/next of kin was not required to participate in this study in accordance with the national legislation and the institutional requirements.

## Author contributions

Study concept and design: KA-K, SP, AS. Executed the study: SP, AS, PA, NS, SA-S, FA-D. Statistical analysis: SP. Drafting the article: KA-K, AS, SP. Critical revision of the article for important intellectual content, writing of the article, and approval of the final version: KA-K, SP, AS, PA, NS, SA-S, FA-D. All authors contributed to the article and approved the submitted version.
